# Proceedings of international symposium of trends in radiopharmaceuticals 2023 (ISTR-2023)

**DOI:** 10.1186/s41181-023-00224-0

**Published:** 2023-11-10

**Authors:** Amirreza Jalilian, Clemens Decristoforo, Melissa Denecke, Philip H. Elsinga, Cornelia Hoehr, Aruna Korde, Suzanne E. Lapi, Peter J. H. Scott

**Affiliations:** 1 Division of Physical and Chemical Sciences, International Atomic Energy Agency, Vienna, Austria; 2 Department of Nuclear Medicine, Medical University Innsbruck, Innsbruck, Austria; 3 Department of Nuclear Medicine and Molecular Imaging, University Medical Center Groningen, Groningen, The Netherlands; 4 Life Sciences Division, TRIUMF, Vancouver, Canada; 5 Departments of Radiology and Chemistry, O’Neal Comprehensive Cancer Center at UAB, University of Alabama at Birmingham, Birmingham, AL USA; 6 Department of Radiology, University of Michigan, Ann Arbor, MI USA

## Abstract

The International Atomic Energy Agency (IAEA) held the 3rd International Symposium on Trends in Radiopharmaceuticals, (ISTR-2023) at IAEA Headquarters in Vienna, Austria, during the week of 16–21 April 2023. This procedural paper summarizes highlights from symposium presentations, posters, panel discussions and satellite meetings, and provides additional resources that may be useful to researchers working with diagnostic and therapeutic radiopharmaceuticals in the academic, government and industry setting amongst IAEA Member States and beyond. More than 550 participants in person from 88 Member States attended the ISTR-2023. Over 360 abstracts were presented from all over the world by a diverse group of global scientists working with radiopharmaceuticals. Given this group of international radiochemists is unique to ISTR (IAEA funding enabled many to attend), there was an invaluable wealth of knowledge on the global state of the radiopharmaceutical sciences present at the meeting. The intent of this Proceedings paper is to share this snapshot from our international colleagues with the broader radiopharmaceutical sciences community by highlighting presentations from the conference on the following topics: *Isotope Production and Radiochemistry, Industrial Insights, Regional Trends, Training and Education, Women in the Radiopharmaceutical Sciences, and Future Perspectives and New Initiatives.* The authors of this paper are employees of IAEA, members of the ISTR-2023 Organizing Committee and/or members of the EJNMMI Radiopharmacy and Chemistry Editorial Board who attended ISTR-2023. Overall, ISTR-2023 fostered the successful exchange of scientific ideas around every aspect of the radiopharmaceutical sciences. It was well attended by a diverse mix of radiopharmaceutical scientists from all over the world, and the oral and poster presentations provided a valuable update on the current state-of-the-art of the field amongst IAEA Member States. Presentations as well as networking amongst the attendees resulted in extensive knowledge transfer amongst the various stakeholders representing 88 IAEA Member States. This was considered particularly valuable for attendees from Member States where nuclear medicine and the radiopharmaceutical sciences are still relatively new. Since the goal is for the symposium series to be held every four years; the next one is anticipated to take place in 2027.

## Background

*Overview of ISTR-2023* The IAEA held the 3rd International Symposium on Trends in Radiopharmaceuticals, (ISTR-2023), in IAEA Headquarters in Vienna, Austria during the week of 16–21 April 2023. The event was organized by the *Radiochemistry and Radiation Technologies Section of IAEA* and provided scientists and other professionals working in the production of radioisotopes and radiopharmaceuticals with an international forum to discuss the most recent developments and challenges. During the event, two recent IAEA publications in the field were also announced, “*Theranostic Copper-64 Radiopharmaceuticals*” and “*Preclinical Studies with Radiopharmaceuticals*”, as outcomes of IAEA Coordination Research Projects and technical activities in recent years, respectively.

**Fig. 1:**
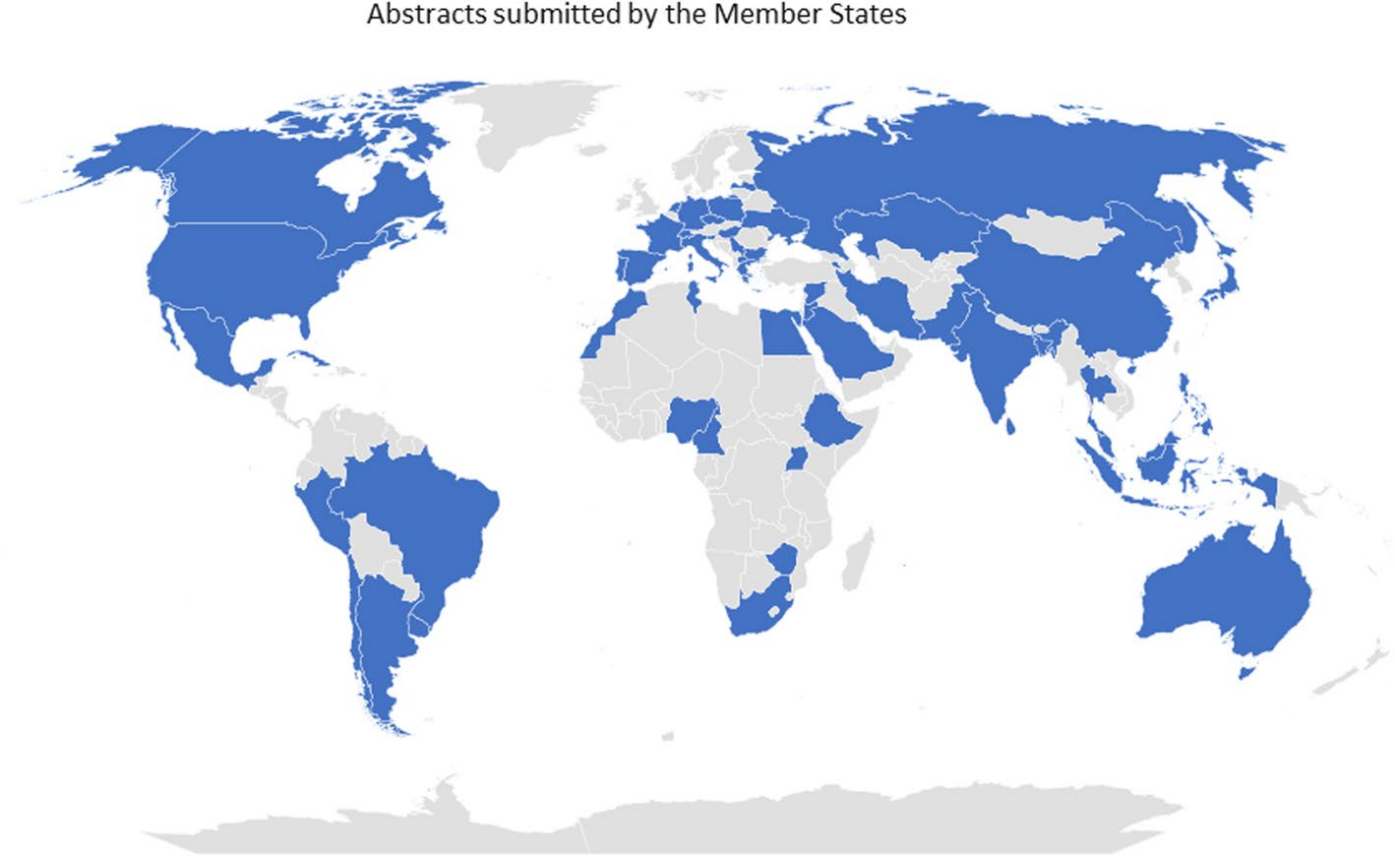
Geographical distribution from which abstracts were submitted by Member States for ISTR-2023

**Fig. 2:**
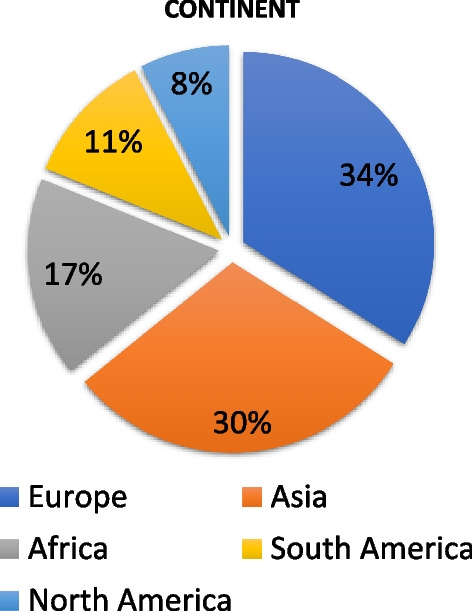
Distribution of continents from where abstracts were submitted by Member States for ISTR-2023

More than 550 participants in person from 88 Member States attended the ISTR-2023 Conference over the course of five days (Fig. 1). Three international organizations, 28 physical exhibitors and one virtual exhibitor, and 368 virtual observers using the website link, attended the event. Over 360 abstracts were presented from all over the world, with about 1/3 coming from Asia and 1/3 from Europe (Fig. 2). Participants at the Symposium represented a diverse group of global scientists working with radiopharmaceuticals. For example, more than 220 posters (160 in person and 66 virtually) were presented by young scientists, while 40% of all participants were women.

One of the main outcomes of ISTR-2023, and what makes IAEA Conferences unique, is the large presence of participants coming from lower income countries. More than 140 participants received grants from IAEA to attend the Symposium. The fact that there is no registration fee is another important factor for attracting a large number of attendees. Thus, bringing this group of international radiochemists and imaging scientists together is considered unique to ISTR-2023 because IAEA funding enabled many to attend from parts of the world where attendance at some of the other large conferences is impractical (e.g. Annual Meetings of the European Association of Nuclear Medicine or the Society of Nuclear Medicine and Molecular Imaging, and the International Symposium on Radiopharmaceutical Sciences). Given this, there was an invaluable wealth of knowledge on the global state of the radiopharmaceutical sciences present at the meeting, and ISTR-2023 organizers paid significant attention to allow time for networking, to facilitate cordial interactions among global participants overcoming various barriers during the event.

The intent of this Proceedings paper is to share this snapshot captured by international colleagues with the broader radiopharmaceutical sciences community by highlighting presentations from the conference and depicting the trends in the field in a nutshell. These outcomes make ISTRs unique, and the valuable all-inclusive global radiopharmaceutical community present at ISTR-2023 indirectly strengthens and supports sustainability and growth in the radiopharmaceutical sciences. It is advocated that professional organizations, scientific societies and different stakeholders from IAEA Member States related to radiopharmaceuticals continue to extend their support to the IAEA for organization of the next meeting in the ISTR series, which is anticipated to take place in 2027.

**Fig. 3:**
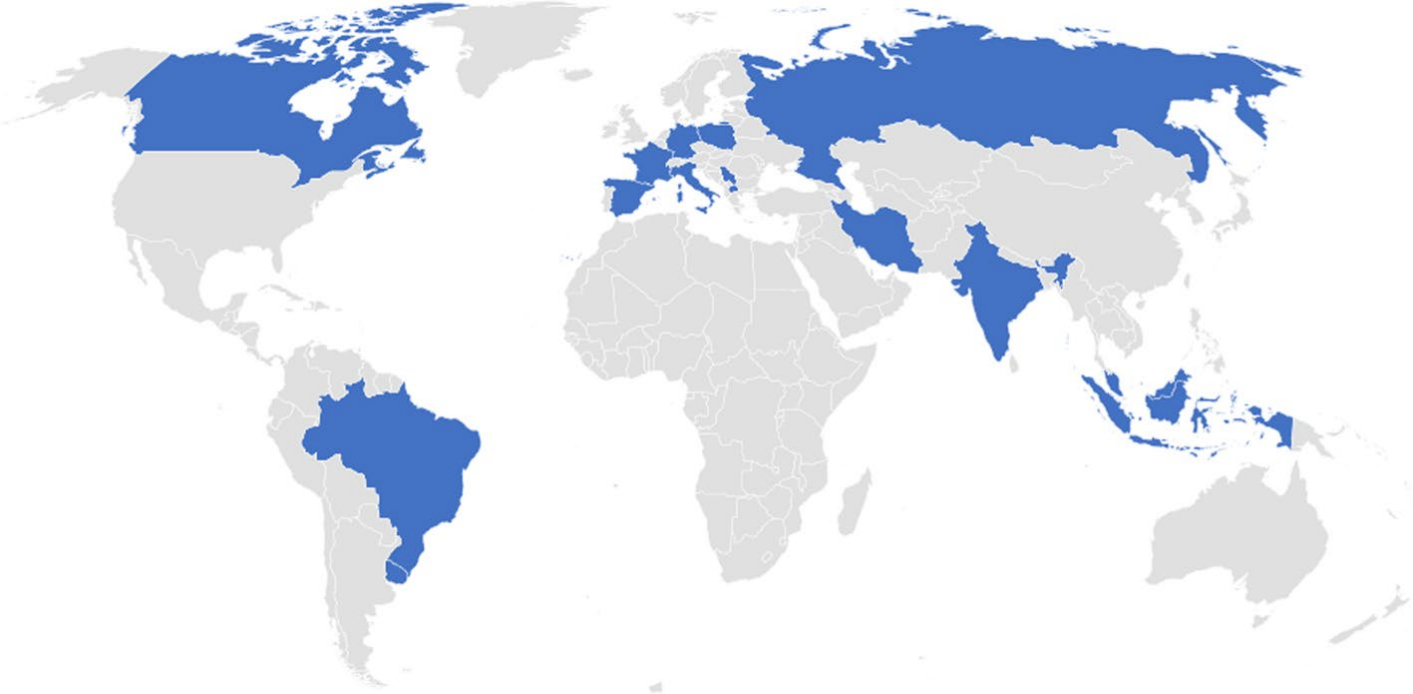
Geographical distribution of young investigators that submitted applications to the Young Investigator award by Member States for ISTR-2023

Participation of young scientists in oral and poster presentations was robust during the week, and Young Investigators Awards for the best posters were granted to five selected scientists (80% female awardees) from a shortlist of applicants from around the world (Fig. 3). A mentoring side event for the young generation of scientists was held to encourage a new wave of radiopharmaceutical scientists.

**Fig. 4:**
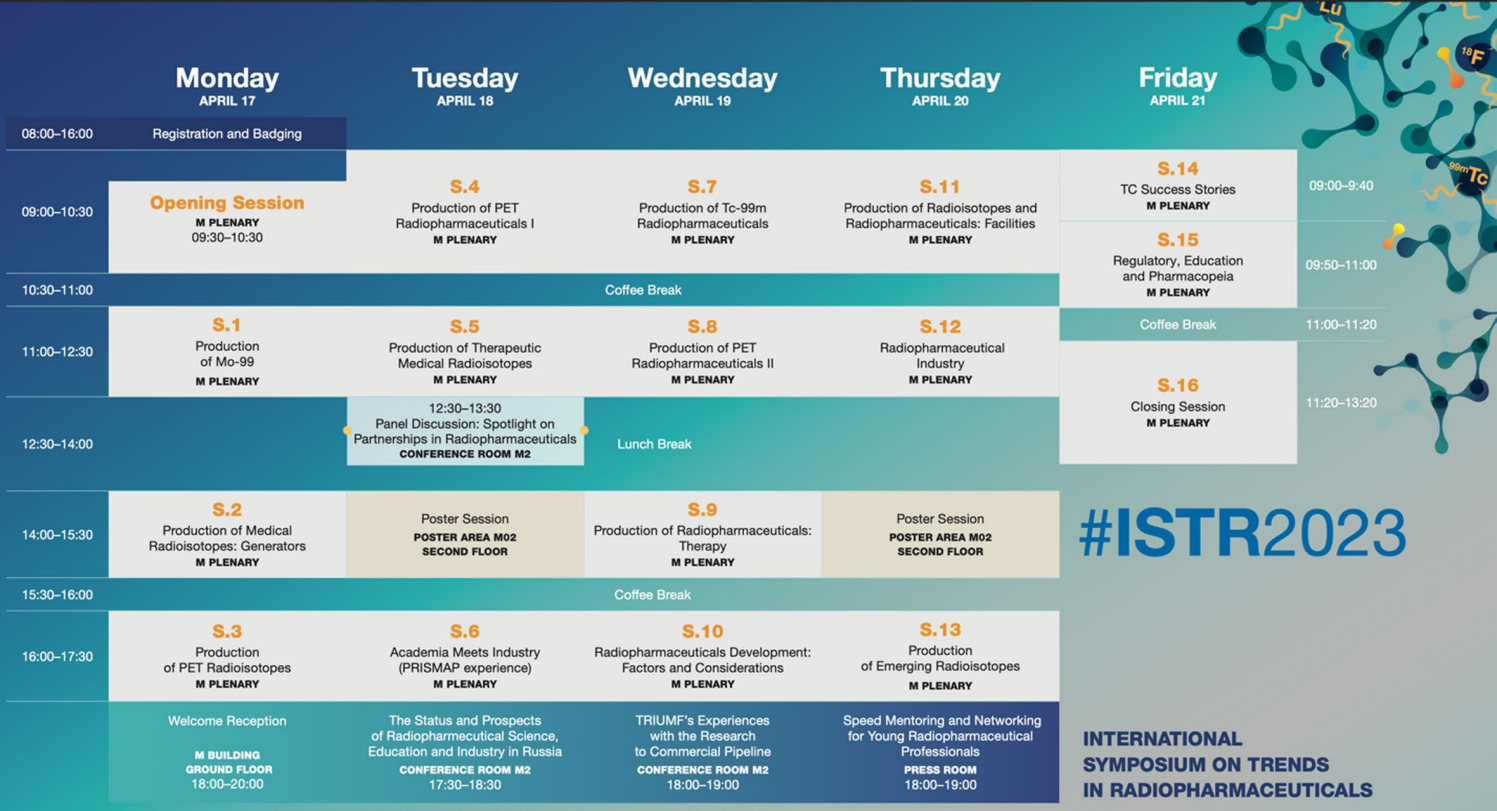
ISTR-2023 Program

The program spanned 4.5 days (Fig. 4) and the scope of the Symposium included, but was not limited to, the following topical areas:

• Production of diagnostic (Positron Emission Tomography (PET) and Single Photon Emission Computed Tomography (SPECT)), therapeutic and theranostic medical radioisotopes;

• Production of radionuclide generators;

• Production of diagnostic, therapeutic and theranostic radiopharmaceuticals;

• Production and quality control of alpha emitter radiopharmaceuticals;

• Research and development related to the production of medical radioisotopes and radiopharmaceuticals;

• Quality control and quality assurance of medical radioisotopes and radiopharmaceuticals;

• Preclinical evaluation of radiopharmaceuticals including data needed for approvals, case studies including animal/human compliance and statistics;

• Dosimetry for new radiopharmaceuticals (both imaging and therapeutic agents);

•Good Manufacturing Practices (GMP) and other guidelines for production of medical radioisotopes and radiopharmaceuticals;

• Design of radiopharmacy (industrial, hospital and centralized) facilities

• Regulatory aspects related to radiopharmaceuticals;

• Accelerators for radioisotope production (choice, design etc.);

• Radiopharmacy Chapter in pharmacopoeias;

• Women in radiopharmaceutical sciences, trends, challenges, and future;

• Education, including e-learning, certification and training methodologies for professionals involved in radiopharmaceutical sciences;

• Introduction to the latest innovations in the radioisotopes and radiopharmaceutical industry.

Given all of these metrics, and a large number of abstracts (> 360) presenting high-quality radiopharmaceutical research from around the world, ISTR-2023 was a success. This paper summarizes highlights from ISTR-2023 presentations, posters, panel discussions as well as satellite meetings, and provides additional resources that may be useful to researchers working with diagnostic and therapeutic radiopharmaceuticals in the academic, government, and industry setting. Given the scope of the conference, it is not possible to provide comprehensive information on selected presentations or highlight every abstract from the meeting. For more information on all of the science presented at ISTR-2023, readers are referred to the Book of Abstracts (see *Availability of Data and Materials* for the link).

### Satellite sessions

In addition to oral and poster sessions around the various core topics, a variety of satellite events also took place. For example, during the symposium an interesting *Panel Discussion* on “Partnerships in Radiopharmacy” was held, chaired by Ms Najat Mokhtar, the IAEA Deputy Director General of Department of Nuclear Sciences and Applications and attended Mr. Luther Gwaza from the World Health Organization (WHO), Mr Excellency Ambassador Almeida e Sousa (the Ambassador of Portugal), and other stake holders such as the Organisation for Economic Co-operation and Development-Network Environment (OECD-NE), Urenco and the most recent IAEA Collaborating Center in radiopharmaceutical sciences in China.

A session was held that presented several success stories of the important IAEA Technical Cooperation Services to Member States, with one example from each geographical region (Asia–Pacific, Europe, Africa and Latin America).

During the Symposium, two technical side events were also held from leading Member States in the field of radioisotope and radiopharmaceutical production. In order to strengthen the communications between Member States and industries and to introduce the latest technological developments, a session on “*Radiopharmaceutical Industries*” was held with presentations from 15 companies. The European medical radioisotopes programme PRISMAP [https://www.prismap.eu], an infrastructure consortium supported by the EU, also held a session with presentations of regional collaboration on innovations on medical radioisotopes production.

## Main text

### Isotope production and radiochemistry

#### Diagnostic radionuclides

***Technetium-99m***

Several groups reported on the production and radiochemistry of ^99m^Tc illustrating that in many countries ^99m^Tc planar and SPECT imaging are still the work horses of nuclear medicine. These talks included updates on availability of ^99^Mo supply and generators, developments on ^99^Mo generator production, other supply lines of ^99m^Tc and radiochemistry studies towards new ^99m^Tc radiopharmaceuticals.

A European perspective on the supply of ^99^Mo was presented by Bernard Ponsard and included an update on the conversion from highly enriched uranium (HEU) to low enriched uranium (LEU) targets at the BR2 and other reactors. Excitingly, all fission ^99^Mo is currently produced from LEU targets with a current estimated global demand of 9000 Ci ‘6d-calibrated’ ^99^Mo per week. A security of supply (SoS) working group continues to meet regularly to coordinate the supply chain and an emergency response team has been formed to address related issues. Overall there is currently efficient coordination of the research reactors operating periods and good logistics but concern remains as several research reactors will reach their ‘end of life’ in the next decade.

Non-fission ^99^Mo lines of supply are also coming online leading to a diversification of sources. This includes photonuclear production as reported by Takahiro Tadokoro from Hitachi in Japan. In proof-of-concept studies, targets composed of MoO_3_ powder yielded radionuclidically pure ^99^Mo in agreement with theoretical yields. The team also proposed to use photonuclear production as a route towards other isotopes including ^67^Cu and ^225^Ac.

Chris Horne from Laurentis/ BWX Technologies (BWXT) in Canada reported on the development of neutron capture based production of ^99^Mo using commercial CANDU-type power reactors. This technology has been installed in one reactor with the opportunity for expansion.

Paul Schaffer from the TRIUMF group (Canada) reported on continued efforts for cyclotron production of ^99m^Tc. High power ^100^Mo targets have been developed capable of producing yields of 19 Ci/target and a 60 patient clinical trial has been completed (Bénard et al. 2014). The impurity profile was heavily dependent on the isotopic enrichment of the starting material and critical Tc radionuclidic purity is determined by the content of ^92−97^Mo (Celler et al. 2011). The clinical trial concluded that radiotracer biodistribution and clinical image quality obtained with cyclotron-produced ^99m^Tc is equivalent to the image quality obtained with generator-produced ^99m^Tc and that cyclotron-produced ^99m^Tc is safe for clinical use in human subjects.

Development of methods for production of ^99m^Tc generators in small batches (< 30 generators per batch) was detailed by Aleksandar Vukadinović from Vinča" Institute of Nuclear Sciences, Belgrade, Serbia. This included automation of the chemistry process, dispensing and QC process in compliance with GMP requirements and in an affordable manner. In a similar fashion, Montaña et al. from Cuba reported on the larger scale production of ^99m^Tc generators in a new dedicated facility and supply to the regional market. New ^99m^Tc kits are also under development from this group.

Work on new generator types including developments on gel generators in the Republic of Kazakhstan was presented by Ye Chakrova. These gel generators are in production regularly and make use of low specific activity ^99^Mo produced from neutron capture (1.5–2.3 Ci/g). Importantly, the resulting ^99m^Tc also meets standards set by the European Pharmacopoeia. The group also reported that the use of molybdenum enriched to at least 95% of ^98^Mo increases the specific activity of ^99^Mo up to 3.5 times, depending on target geometry, with a corresponding increase of the generator activity.

^99m^Tc radiopharmaceuticals presented included cefepime as an agent for imaging infection in a presentation from Abdelmjid Aiboud from Centre National De L'énergie, Des Sciences Et Techniques Nucleaires (CNESTEN), Morocco. This leverages a radiolabeled version of a broad spectrum antibiotic for the detection of infections using a previously reported hydrazinonicotinamide (HYNIC) chelator and tricine for ^99m^Tc complexation. The team reported on studies to confirm the antimicrobial activity of the conjugate as well as optimized radiolabeling using a tine chloride reduction method. Radiochemical yields > 50% were achieved with > 90% stability out to 3 h. The team plans to move this compound forward into preclinical imaging studies.

#### Positron emitters

The variety of positron emitters available continues to expand and enables a new generation of imaging radiopharmaceuticals.

^18^F is still the most widely used isotope for positron emission tomography (PET). Philip Elsinga from University Medical Center Groningen, Netherlands, provided an excellent update on new chemistry for the development of ^18^F radiopharmaceuticals including the benefits of late-stage fluorination techniques and Al^18^F chemistry. Al^18^F chemistry was also discussed in a presentation from Eduardo Savio from Centro Uruguayo de Imagenología Molecular in Uruguay. This included the radiochemistry development of an Al^18^F PSMA agent and images from a first in human clinical trial (dos Santos et al. 2020). Similarly, Peter Scott from the University of Michigan, USA, gave an overview of the current state of the art for radiochemistry with ^11^C. The presentation highlighted some of the benefits of working with short-lived ^11^C (t_1/2_ = 20 min), noting that multiple scans can be conducted during a single patient visit and that molecules can be radiolabeled without changing the structure (cs. [^11^C]choline and [^18^F]fluoromethylcholine) simplifying, for example, clinical translation. Standard production of [^11^C]CO_2_ was covered, as well as its conversion to common synthons (e.g. [^11^C]MeI, [^11^C]MeOTf) for standard ^11^C-labeling as well as emerging strategies such as ^11^C-fixation, ^11^C-carbonylation, copper-mediated radiocyanation, including new radical approaches.

A presentation on the status of ^68^Ga generators from Frank Rösch from Johannes-Gutenberg-Universität in Germany highlighted challenges and considerations in both generator design and the implementation of direction production using zinc targets on a cyclotron. Additionally, this presentation discussed advances in instant kit labeling and the development of new chelators. Cyclotron production of ^68^Ga was also discussed by Luna Barua from the Department of Atomic Energy, India, using a solid target system. This group also discussed onboarding of [^68^Ga]Ga-DOTATATE using this production method. Tamer Sakr from the Egyptian Atomic Energy Authority also presented on the development of ^68^Ga-WSSF-DOTA-Bombesin nanoparticles with promising early preclinical results. Shuichi Shiratori from Mahidol University in Thailand presented on the group’s work developing a ^68^Ga-tri-γ-glutamic acid polypeptide. This included synthesis, high yield radiolabeling and QC parameters.

The application of ^89^Zr as a radionuclide for the imaging of molecules with longer biological half-lives continues to grow, as underpinned in a presentation on high current sintered targets and an automated production system by Emiliano Cazzola from Sacro Cuore Hospital, Negrar, Italy. This team also reported on an automated synthesis strategy for human use production of radiolabeled antibodies. Katerina Kolevska from the University Institute for Positron-Emission Tomography in North Macedonia presented a feasibility study for the establishment of ^89^Zr production in North Macedonia, including technical and economic analysis. Jeong Hoon Park from Korea presented recent work on their group on the scale up of ^89^Zr production and radiolabeling techniques for nanomaterials. They also reported a novel technique using ^89^Zr Cherenkov radiation coupled with a nanocomposite to induce a therapeutic effect (Choi et al. 2023).

Several presentations highlighted radio-Cu production using solid or liquid targets. Alexandra Fonseca from Coimbra presented her work focused on the use of ^61^Cu and ^64^Cu using liquid targets (do Carmo et al. 2017). Expanding on the group’s prior work, this presentation reported on steps towards recycling of the enriched target materials and the importance of trace metal grade materials and reagents. Miguel Avila-Rodriguez from the Universidad Nacional Autónoma de México, Mexico, gave an update on ^64^Cu production and applications at the National Autonomous University of Mexico. This included a robust method for production and exciting results from a clinical trial imaging [^64^Cu]CuCl_2_ in cancer patients. This presentation also reported on promising early results on PET imaging of infection with [^64^Cu]Cu-DOTA-UBI and other early phase clinical trials. ^64^Cu imaging applications were also presented by Behrouz Alirezapour from NSTRI, Iran. A clinical trial with ^64^Cu-Trastuzumab illustrated promising results in HER2-positive breast cancer. This presentation also highlighted preclinical results with several ^64^Cu peptides for imaging applications in oncology.

Jonathan Engle from the University of Wisconsin, USA, presented recent developments in the production of radiometals using a low energy cyclotron. This included work on new imaging agents for theranostic pairs including ^55^Co and ^43/44g^Sc as imaging analogue for ^58m^Co and ^47^Sc. Production of ^44^Sc was also highlighted in a presentation by Nicholas van der Meulen from Paul Scherrer Institute (PSI), Switzerland. This included development of calcium targets, purification chemistry, and preclinical imaging results with ^44^Sc-PSMA-617. This team also reported on a first in human study with this compound. Suzanne Lapi from the University of Alabama at Birmingham, USA, also presented work on the development of matched pairs for theranostic developments including production of ^43^Sc from titanium targets and ^203^Pb as an imaging analogue for the therapeutic ^212^Pb.

Overall these sessions show a continued global interest in the radiochemistry of organic and radiometal PET isotopes with an expansion of production sites capable of expanding the availability of longer lived isotopes such as ^89^Zr and ^64^Cu. New isotopes suitable for the development of the imaging components for theranostic pairs are also under development.

#### Therapeutic isotopes

In addition to traditional imaging isotopes, there is a strong trend towards the production of therapeutic isotopes, as evident by the large number of sessions and individual talks. These encompass beta emitters from the established ^131^I and ^177^Lu to the more up and coming, e.g. ^161^Tb. Alpha emitters have attracted strong interest in recent years, foremost ^225^Ac and ^211^At, due to their potent treatment efficacy as they exhibit a large linear energy transfer (LET). Finally, Auger emitters are being produced in several settings, as they hold the promise of improved therapeutic outcome due to their very short range of only a diameter of a cell.

#### Beta emitters

Established beta emitters that are in clinical use for several years already are of great importance to the community. Among these, Tladi Moloto from NTP Radioisotopes SOC Ltd, South Africa, discussed production of ^131^I via the nuclear reactor operated by the South African Nuclear Energy Corporation (NECSA) as well as its global supply issues and mitigation points. ^177^Lu is another well-established beta emitter, and its production at the McMaster Nuclear Reactor (MNR) in Canada via the ^176^Lu(n,γ)^177^Lu was presented by Andrea Armstrong from McMaster University. ^90^Y has long been clinically used in microspheres to treat liver cancer. Jie Gao and colleagues from the China Institute for Radiation Protection (CIRP) have developed ^90^Y carbon microspheres, in addition to the two commercially available systems, and are currently conducting clinical trials.

^188^Re via the ^188^W/^188^Re generator can also be used for beta therapy. ^188^W is produced by irradiating ^186^W targets in nuclear reactors. The history and availability of the generator, as well as its production by POLATOM in Poland and SCK-CEN in Belgium was presented by Renata Mikołajczak from the National Center for Nuclear Research, Poland.

Finally, the emerging beta emitter ^161^Tb was discussed by Nicholas Van der Meulen from PSI, Switzerland, including approaches for its production at PSI. ^161^Tb is similar to ^177^Lu but emits in addition a substantial number of conversion and Auger electrons, potentially increasing the therapeutic effect.

#### Alpha emitters

Cathy Cutler from Brookhaven National Lab (BNL), USA, provided an update from the Department of Energy (DOE) in the USA on its large network of production sites either developing production capabilities or producing and supplying alpha emitters for therapy. These include ^225^Ac, ^211^At, ^212^Bi, ^213^Bi, ^212^Pb, ^223^Ra, ^226^Th and ^227^Th. For example, ^225^Ac is being produced with proton irradiation at BNL and Los Alamos National Laboratory (LANL), and development of the production with electrons via a photonuclear production mechanism is underway at Argonne National Laboratory (ANL). This is in addition to their production from the decay of existing ^229^Th stock.

DOE reactors are producing ^227^Ac, ^228^Th and ^229^Th. ^212^Bi is supplied via a generator system from the parent isotope ^212^Pb, which is being produced as a decay product from ^228^Th. Alternative production routes explored by the DOE are ^226^Ra(γ,n)^225^Ra → ^225^Ac, ^226^Ra(p,2n)^225^Ac, and ^226^Ra(3n, γ)^229^Ra → ^229^Ac → ^229^Th.

The production and distribution of ^211^At, while challenging due to its relatively short half-life of only 7.2 h, is also of great interest in Japan where Kohshin Washiyama from Fukushima Medical University, Japan, reported it is produced via the ^209^Bi(α,2n)^211^At reaction at five different production sites for more than 20 end users. The same production route is chosen in Europe through the Network for Optimized Astatine-labeled Radiopharmaceuticals (NOAR) COST Action. While there are currently only two active production sites at Copenhagen University in Denmark and Arronax in France, Jean-Francois Gestin, from ISOTOP4LIFE, reported that several additional sites will start production soon.

^225^Ac is often used as a generator isotope for ^213^Bi in its decay chain. In this case, ^225^Ac is a contamination in the end production that needs to be minimized as reported by Lukáš Ondrák on behalf of a collaboration between the Joint Research Centre in Karlsruhe, Germany, and the Czech Technical University in Prague.

Another interesting alpha emitter under development is ^149^Tb. Nicholas van der Meulen from PSI, Switzerland, reported that a collaboration between PSI and ISOLDE (Isotope Separation OnLine) facility at CERN is currently producing ^149^Tb by proton-induced spallation of a tantalum target at PSI, followed by an online isotope separation process at ISOLDE. ^149^Tb is of special interest as it forms theranostic pairs with ^152^Tb for PET, ^155^Tb for SPECT and ^161^Tb can be used for therapy.

Finally, several alpha emitters are available via the PRIMSAP consortium in Europe, for example ^149^Tb, ^211^At, ^223^Ra, ^225^Ac, and ^227Th^ as reported by Thierry Stora, an academic researcher from Conseil Européen pour la Recherche Nucléaire (CERN).

#### Auger emitters

Several Auger emitters can be produced on lower energy, medical cyclotrons. Among those, the production of ^58m^Co from solid targets was reported by Jonathan Engle at the University of Wisconsin Cyclotron lab. Also, the production of ^119^Sb and ^197^Hg from solid targets was reported by Cornelia Hoehr and Valery Radchenko, from the group at TRIUMF, Canada, with the ^197^Hg then being incorporated into gold nanoparticles. In addition, ^103^Pd from a liquid target route has been reported by TRIUMF as well.

### Radiopharmaceutical development: factors and considerations for clinical translation, including regulatory aspects

The clinical translation of radiopharmaceuticals is a challenging process to overcome both technical hurdles and finally to meet regulatory requirements. In this session, novel developments to facilitate and accelerate radiopharmaceutical developments from bench to bedside as well as new applications were presented and the regulatory challenges addressed.

Peter Scott from the University of Michigan, USA, showed how in silico methods can help to support radiochemists in selecting lead compounds and radiotracer design and synthesis. In CNS radiotracer development for PET, failures in the development are common mainly due to insufficient radiochemical yields, poor brain uptake, high non-specific binding or other reasons. Artificial Intelligence (AI) can be applied to help overcome certain challenges in this context. Whereas high throughput screening traditionally is time consuming, resource intensive, and may miss obvious trends, computational approaches may solve some of these problems. By assembling control libraries of well characterized tracers from literature and defining parameters of success and failure, artificially generated weighing scores may predict successful candidates (Jackson et al. 2021). Another approach is to apply machine learning for radiochemistry thereby connecting method development with the application (Jackson et al. 2022). Data from literature can be applied on a model, which then can be trained related to substrate and reaction featurization, to predict radiochemical conversion for a particular reaction (Jackson et al. 2022; Webb and Scott 2021). Overall, AI holds promise to change the definition of lead compounds or radiotracer design and synthesis, enabling an entirely digital discovery paradigm in radiopharmaceutical development for certain applications.

Another field of development is the availability and increasing use of new radioisotopes in preclinical research. Suzanne Lapi from University of Alabama at Birmingham, USA, presented data on ^45^Ti, a PET radionuclide with a half-life of 3.08h, which can be produced in low to medium energy cyclotrons from natural scandium (Chaple et al. 2020). Due to its longer half-life as compared to ^68^Ga, it can be shipped and in a recent proof of principle ^45^Ti targeted tracers for PET imaging of PSMA were reported (Chaple et al. 2022). Another emerging PET radionuclide with extended half-life of 5.6d is ^52^Mn, which can be cyclotron produced from natural Chromium (Pyles et al. 2021). ^52^Mn showed promising results for labelling trastuzumab and use for imaging up to 14d p.i. in a mouse tumor model. These examples showed that a wide variety of half-lives, imaging characteristics and chemistries leads to a unique toolbox for the development of new nuclear medicine imaging and therapeutic agents and asks for extensive collaboration between chemists, biologists, physicists, physicians and technologists.

Small animal imaging, however, can not only be used for radiopharmaceutical development as such, but also in a much wider context of drug development. Oliver Langer from the Medical University of Vienna, Austria, presented the potential to use small animal imaging to improve pharmacokinetic (PK) analysis of drugs using imaging-based PK analysis in mice for translation into the human setting, which is facilitated by the use of radiotracers and the concept of Microdosing. Based on the example of Ciprofloxacin, which shows renal transport mediated drug-drug interactions, it was shown how small animal PET can help to find the cause for specific drug interactions. Using prototypical renal anion and cation transporter inhibitors it could be shown that decreased renal excretion, leading to potential exacerbation of therapeutic efficacy or side effects, was caused by inhibition of basal uptake transporters and apical efflux transporters in kidney proximal tubule cells (Hernández-Lozano et al. 2023). This example shows the potential for small animal PET to assess PK by whole body imaging with appropriate modelling and establishing links between PK and PD as well as drug-drug interactions. Therefore, it is a very useful translational tool also in planning and supporting Phase 0 studies in humans allowing facilitation of drug development by abbreviated non-clinical safety packages.

Finally, the regulatory environment of radiopharmaceuticals poses a major challenge in the clinical translation. Clemens Decristoforo from the Medical University of Innsbruck, Austria, provided an overview of major steps in radiopharmaceutical development and where non-clinical data generation is required to provide supportive evidence for the expected human in vivo behaviour, particularly related to safety and efficacy. In relation to the radioactivity, an estimate of the expected human radiation dose has to be established, usually by preclinical dosimetry studies. To generate data to predict pharmacology and pharmacokinetics, *in-vitro* and *in-vivo* studies are required, which may be facilitated and improved by using imaging approaches. New regulatory guidance allows a more risk-based approach towards toxicity testing of the “cold” molecule to be applied in humans. IAEA has provided support for the technical conduct of such preclinical studies (International Atomic Energy Agency, 2021), and also has recently supported an effort to develop guidance on the practical considerations for navigating the regulatory landscape of non-clinical studies for clinical translation of radiopharmaceuticals (Korde et al. 2022), which summarizes guidance documents and guidelines, putting them into the context of radiopharmaceutical development.

Overall, this session provided an excellent insight into the prospects and developments for the clinical translation of novel radiopharmaceuticals, exemplified by the use of artificial intelligence and novel radionuclides, and the use of radiotracers for conventional drug development, but also addressed the challenges of the regulatory framework that requires adaptation to cope with innovation in radiopharmaceutical sciences.

### Industrial insights

Olga Valzdorf from the Rosatom State Corporation, Russia, presented an overview of registered radiopharmaceuticals as well as trends in clinical trials. Rosatom State Corporation has the largest capacity in the world, exploiting eight reactors. In order to prioritize isotope production, they argue it is necessary to have an overview of the isotopes and molecules being utilized in multinational clinical trials, as well as radiopharmaceutical indications, doses, timelines, infrastructure conditions and other information that may influence current and future demand for isotope products. This is an important idea where additional partnership with IAEA might be useful.

Maria Gonzalez reported on the efforts of INVAP, a state company founded in Argentina in 1976 that is involved in projects all over the world. Within the nuclear area, INVAP participates in a variety of projects including radioisotope production plants, radiopharmaceutical production plants, nuclear medicine centers, and measurement systems for radioisotope emission. For example, molybdenum-99 production plants in Egypt and India, research reactors in Australia, Peru, Algeria, Argentina, Brazil, the Netherlands, and nuclear medicine centers for radiopharmaceutical production (e.g. [^18^F]FDG, [^11^C]choline and [^13^N]ammonia) in Argentina and Bolivia, are some of the projects that INVAP has undertaken.

### Regional trends

#### General considerations

**Fig. 5:**
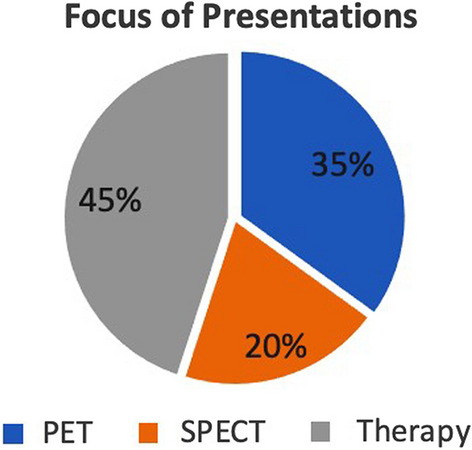
Focus of the Presentations

Nuclear medicine is a field undergoing a lot of changes. In low income nations, the technology is still being established, in large part due to the efforts of IAEA, while in some of the more technologically advanced member states it is evolving from a research technique to a standard of care, particular with the emergence of theranostics. Somewhat surprisingly, but perhaps reflective of this latter point, nearly half of the presentations at ISTR-2023 were focused upon production and applications of radiotherapeutics (Fig. 5). Another consideration is that the long half-lives of some therapeutic radionuclides (e.g. ^225^Ac t_1/2_ = 9.9 d, ^177^Lu t_1/2_ = 6.6 d) likely make them more available to places without local production capabilities.

Given that approximately 80% of global nuclear medicine diagnostic studies still consist of SPECT scans, and that this percentage is higher in Member States that have access to ^99^Mo/^99m^Tc generators and SPECT scanners but that do not yet have established networks of more costly cyclotrons and PET scanners, it was also interesting that more presentations covered production and/or application of PET radionuclides than SPECT. However, a deeper dive into the PET abstracts reveals that most are using either ^68^Ga from generators or long-lived PET radionuclides like ^64^Cu (t_1/2_ = 12.7 h) and ^89^Zr (t_1/2_ = 78.4 h) that can be shipped from centralized cyclotron facilities. These diverse radionuclide sources, that do not require an expensive and complex cyclotron radiochemistry facility on site like classical PET studies (e.g. using ^11^C, ^13^N, ^15^O or ^18^F), appear to be improving access to PET amongst Member States.

When considering classical cyclotron-produced PET radionuclides dependent upon access to local accelerator infrastructure, strides are being made as described throughout these proceedings. However, there are still many groups in IAEA Member States which have limited or non-existent access. Indeed, when Peter Scott gave an overview of the current state of the art for radiochemistry with ^11^C (t_1/2_ = 20 min) and asked the audience, by show of hands, “who works with carbon-11?”, only about 5–10 people answered in the affirmative (~ 1% of the audience). This is in line with the low number of ^11^C abstracts submitted to ISTR-2023 (~ 2–3 from Europe and South America). Conversely, a few dozen abstracts included work with ^18^F, presumably being obtained by facilities with reasonable proximity to commercial nuclear pharmacies from which the isotope can be shipped, given the longer half-life of ^18^F (t_1/2_ = 110 min) compared to ^11^C.

As use of nuclear medicine around the world continues to increase, numerous approaches to training and education are being implemented which are described in a dedicated section below. There also continues to be efforts to establish regulations around the world for radiopharmaceuticals, and a continued push for harmonization between different jurisdictions. Ramón Alvarez from the Office for Environment and Safety Regulation, Nuclear Safety Division, in Cuba presented lessons learned on how to obtain better harmonization between the various regulatory authorities involved in the regulation of radiopharmaceutical production. Certain other regional trends were also apparent from the conference, as follows:

#### North America

There were a number of speakers from institutions in North America, many of which have already been described in the preceding sections. Noen Malik and the late Bin Shen from Stanford University, USA, submitted an abstract covering trends in radiopharmaceutical development from bench to clinic, as well as the evolving regulatory environment in the USA. Notably, > 10,000 hospitals worldwide utilize radioactive isotopes produced by cyclotrons / generators and technetium-99 remains the most used isotope, accounting for about 80% of all nuclear medicine work, with some 40 million procedures per year. They also noted that there appears to be a constant increase of > 10% in the number of diagnostic scans employing radiopharmaceuticals every year.

Serge Lyashchenko from the Memorial Sloan Kettering Cancer Centre in New York provided an overview on the regulatory requirements of radiopharmaceutical production, with a focus on the hospital setting in the US.

Terry Campkin from Ontario Power Generation, Canada, gave an update on CANDU role in strengthening the radioisotope supply chain. The reliability of the CANDU reactors and the increasing ways that isotopes can be generated will be a key component to strengthening the radioisotope supply chain for the coming decades, particularly given the rapid growth in utilization of theranostics.

#### South America

There is an abundance of nuclear medicine activity in South America as the technique becomes more routinely used. Barbara Beatriz Dias Rodrigues from the Brazilian National Nuclear Authority (CNEN), for example, discussed use and licensing of ^68^Ge/^68^Ga generators in Brazil. Of the 130 nuclear medicine facilities in the country, 92 of them are licensed to work with ^68^Ga by CNEN. As use of the generators has become more commonplace throughout the country, CNEN has introduced requirements to be met by sites operating ^68^Ga generators, covering radiation protection, shielding, training, recording of dose rate from the generator, and six-monthly reporting requirements. There is also an initiative to nationalize brachytherapy radioactive sources in Brazil, in cooperation with IAEA, which was discussed by Maria Rostelato and colleagues from the Instituto de Pesquisas Energeticas e Nucleares (IPEN-CNEN/SP) in Sao Paulo. Lastly, there were presentations on the need to strengthen clinical trial capabilities in Brazil (Marina Bicalho Silveira from the Nuclear Technology Development Center) as well as on the nations’ maturing regulatory requirements. Alfonso Uruburu and colleagues at the Brazilian Commission of Nuclear Energy (CNEN) discussed the current licensing and safety requirements for radiopharmacies in member countries of the Ibero-American Forum of Radiological and Nuclear Regulatory Bodies. Alessandro Facure and co-workers from Rua General Severiano also discussed new regulatory framework for radioisotopes production and radiopharmacies in Brazil, including CNEN ´s Standard of Safety and Radiological Protection Requirements in Radioisotope Production Facilities with Cyclotron Accelerators and the standard of Safety and Radiological Protection Requirements in Centralized and Industrial Radiopharmacies.

There is also a very active nuclear medicine field in Argentina. For example, Daniela Camporotondi and María Cerizola presented the radioisotope production capabilities for nuclear medicine purposes at the Ezeiza Atomic Centre. The facility houses the RA-3 reactor, which has been in operation since 1967, as well as one of the six cyclotrons in Argentina, with energies of up to 42MeV and currents of 100µA. In addition, the RA-10 reactor, under construction since 2016, will cover 15% of world demand for ^99^Mo/^99m^Tc and shore up national need. A project concept paper from Argentina was also presented for clinical translation of theranostic radiopharmaceuticals. The idea is based on a centralized management system that coordinates the Comisión Nacional de Energía Atómica resources in Buenos that has been designed with assistance from IAEA-Koica. Ana Lopez Bularte and Noemi Nevares from National Atomic Energy Commission Argentina described the goal to provide innovative therapies in the field of nuclear medicine and improve the quality of life of Argentina population. German Rabi from Autoridad Regulatoria Nuclear, also in Buenos Aires, presented the Nuclear Regulatory Authority of Argentina’s regulatory action in the control of ventilation systems during the authorization and inspection processes of the radiopharmaceuticals production facility with cyclotron.

Another report from Richard Ledesma, representing our colleagues in Peru, included improvements to nuclear medicine practices following changes made in response to the Covid-19 pandemic.

#### Africa

Beverley Summers from Sefaco Makgatho Health Sciences University, South Africa, and colleagues provided an update on the current status, opportunities and future directions for radiopharmacy practice in Africa. Overall, the feeling is that current access to radiopharmacy services is still inadequate for the continent. A Society of Radiopharmaceutical Sciences congress (eSRS Africa) took place in 2021, where scientists gathered to discuss the status, gaps and ways to improve radiopharmacy services in African countries. Opportunities described included introduction of current technology, training personnel across the various subfields of radiopharmacy, establishing partnerships with experienced research groups around the world, deploying research models specifically tailored to African perspectives, and sharing experience amongst colleagues. With input from IAEA and SRS, 21 Member States from Africa established the African Association of Radiopharmacy, and the Association will organize the first conference of Radiopharmacy in Africa by the end of 2023.

In a similar vein, Naoual Bentaleb presented ongoing efforts to strengthen radiopharmacy practices in Morocco including establishment of a regulatory framework, a joint master’s programme for French-speaking countries developed by the Faculty of Medicine and Pharmacy of Rabat together with the National Center for Nuclear Energy, Science and Technology (CNESTEN) and IAEA, and their plan to open new nuclear medicine centers across the country, in addition to the existing 25 centers. For example, the CNESTEN has recently obtained marketing authorization of iodine-131 produced using the research reactor Triga Mark II. Two industrial cyclotrons have also been producing short-lived radiopharmaceuticals (mainly FDG) for PET imaging since 2010.

With expanding use of radiopharmaceuticals in Ethiopia (Gaspar 2019), Seble Enyew and Yohannes Lagebo from the Ethiopian Food and Drug Authority (EFDA) reported on the regulatory status of radiopharmaceuticals in Ethiopia. The national nuclear medicine and Radiopharmacy expansion program is under the direct follow up of the central government, which is chaired by the State minister of the Federal Ministry of Health (FMOH) and is under follow up of First Lady of Ethiopia, while day-to-day regulation falls to a number of different groups. The safety standards of radiation are in accordance with IAEA standards. It was also highlighted that the safety, efficacy, and quality of radiopharmaceuticals is to be regulated by the EFDA, whereas the radiation safety of the patient, workers and public as well as the design and building of radiotherapy units will be regulated by the Ethiopian radiation protection authority (ERPA).

Abdulmajeed Ibrahim and Farida Lawal from the Nigerian Nuclear Regulatory Authority drew attention to the inadequate supply of radioisotopes in Nigeria and Sub-Saharan Africa. Nigeria, with a population of over 200 million, is the highest user of radioisotopes in the Sub-Saharan region, and thus the possibilities of investing in isotope production in Nigeria could supply not only the region but the entirety of Sub-Saharan Africa.

There is a more established nuclear infrastructure in South Africa, including a reactor. Maryke Lundie and colleagues at Sefaco Makgatho Health Sciences University talked about ongoing efforts to optimize radiopharmacy services in a multidisciplinary Nuclear Medicine setting in South Africa, and particularly focused on the benefits of involving radiopharmacists as much as possible, while also recognizing the shortage of such personnel and the need to train more. Reflecting this issue, Yohannes Lagebo and Naoual Bentaleb’s report from Addis Ababa, Ethiopia, showing a significant step towards improving radiopharmacy services in Africa is particularly timely.

Other reports from our colleagues in Africa included modeling and assessment of radioactive iodine dispersion inside Egyptian radioisotope production facility (Hesham Elkhatib, EAEA), establishing good radiopharmacy practice in Mauritius as a case study for Africa (Amreeta Mangatha, Nuclear Medicine Department—Mauritius), introduction of a platform for the management, production and distribution of radiopharmaceuticals in Tunisia (Moez Trabelsi, Centre National de Radioprotection), and the regulatory approach to using radiopharmaceuticals in developing countries like Zimbabwe (Luckson Gorondondo, Radiation Protection Authority of Zimbabwe).

#### Asia

Ala’ Khwaj and Abdullah Abu Orouq provided an overview of radiopharmaceutical production in Hashemite Kingdom of Jordan (HKJ). Production began at the King Hussein Medical Centre in 2004, and a new cyclotron facility was installed in 2013. The group also has access to nuclides from a nuclear reactor dedicated for research and training (the Jordan Research & Training Reactor, JRTR), installed in HKJ in 2010 and licensed by the Jordanian regulatory Authority, Energy & Minerals Regulatory Commission (EMRC) in 2017 to produce ^131^I, ^99m^Mo, and ^192^Ir. In December 2018 the team produced the first sample product of Na^131^I, and distributed to all nuclear medicine centers in Jordan after QC testing per EuPh4.

Alfitri Meliana and Siswoto Siswoto from the National Research and Innovation Agency gave an overview of the qualification and certification process for radioisotopes and radiopharmaceutical operators and supervisors in Indonesia. Personnel certification in radioisotopes and radiopharmaceuticals is handled in Indonesia by the BATAN Personnel Certification Body (LSP BATAN), which is currently managed by the Directorate of Competency Development National Research and Innovation Agency. The certificate of expertise is then used as one of the requirements for obtaining a Work Permit (SIB) for Operators of Radioisotope Production Facilities and Radiopharmaceuticals issued by the Nuclear Energy Regulatory Agency (BAPETEN). Only personnel with a license or SIB are able to work as operators of Radioisotope Production Facilities and with radiopharmaceuticals.

Zahra Kousri of the Pakistan Nuclear Regulatory Authority (PNRA) provided an overview of regulatory practices in place in Pakistan to ensure safe and secure production and use of radiopharmaceuticals. The practice is regulated by the PNRA and radiopharmaceutical production facilities follow regulations from the European and US Pharmacopeias. Similarly, there were updates from our colleagues in Bangladesh. Momtaz Waheed from the Bangladesh Atomic Energy Commission (BAEC) updated attendees on Bangladesh’s efforts to advance their national isotope production capabilities and nuclear medicine efforts at 16 nuclear medicine centers under the umbrella of BAEC, in addition to six other private and government run hospitals and eight new nuclear medicine centers poised to start seeing patients soon. In addition, Md. Nahid Hossain from the National Institute of Nuclear Medicine and Allied Sciences (NINMAS) in Bangladesh described experiences and perspectives on the production of radioisotopes at medical cyclotron facilities in Bangladesh. A single cyclotron supplied the entire country until 2020, when BAEC and NINMAS partnered to add a second cyclotron for expansion and redundancy. Given the growth in nuclear medicine in Bangladesh, the government is establishing another three cyclotron facilities around the country within a year.

There is also significant growth in the utilization of radiopharmaceuticals in China. Jianguo Li and co-workers from the China Institute for Radiation Protection (CIRP), a group operating under the National Medical Products Administration (NMPA) for radiopharmaceuticals, described their efforts issuing guidelines for both clinical use and research use of radiopharmaceuticals so as to standardize research, development, and clinical research of diagnostic radiopharmaceuticals in China. In addition to these guidelines, CIRP is also working with multiple agencies including the China Atomic Energy Authority to a Mid and Long-term Development Plan (2021–2035) for Medical Isotopes, which has included preclinical evaluation of radiopharmaceuticals and approval for clinical use.

Adelina Bulos and co-workers from the Philippine Nuclear Research Institute discussed recent radiopharmaceutical utilization profiles in the Philippines. Most of the radiopharmaceuticals used at nuclear medicine centers in the Philippines are currently imported, and the authors also noted the increasing rate of establishment of new nuclear medicine centers. Reflecting this latter point, Ivy Nunez and colleagues from the Philippine Nuclear Research Institute reported on establishment of the Nuclear Medicine Research and Innovation Center for the Development of Emerging PET Radiopharmaceuticals in the Philippines. To date, only privately held facilities are capable of producing cyclotron-based radiopharmaceuticals in the Philippines, making PET imaging limited and expensive. Thus, it is envisioned that this new facility will reduce the cost of cyclotron-based radiopharmaceuticals, with the goal of making them available to lower-income patients in the country.

Additional presentations from Asia included one from Dulanjalee Rajapaksha of the Atomic Energy Board of Sri Lanka on setting up the first radiopharmaceutical production facility in the country, a discussion by Ruben Dallakyan of the Yerevan Physics Institute on medical radioisotope production technology development in Armenia, and a report from Kaisar Turapbay and Marita Jakanova discussing the rapid development of nuclear medicine in the Republic of Kazakhstan, including both diagnostics and therapeutics. Presentations from India included one from Tapas Das on production of ^177^Lu and ^177^Lu-based radiopharmaceuticals at the Bhabha Atomic Research Centre and a discussion from Muhammed Kunnekkadan and colleagues at Molecular Cyclotrons in India on the management of radioactive waste in a cyclotron facility. The latter presentation noted the importance of careful planning at the project stage and the need for sufficient shielded facilities to store long-lived solid waste generated during routine operations.

#### Europe

The biggest update from Europe was on the status of primary radioactivity standards from Steven Judge and colleagues from the International Bureau of Weights and Measures (BIPM) in France. The development of the extension of the BIPM's international reference transfer instrument (ESIR) will help to fill the gap in international comparisons for emerging therapeutic radionuclides, given their increased use for radiotherapy around the world. A presentation from Clemens Decristoforo of the European Association of Nuclear Medicine (EANM) in Vienna discussed the importance of in-house radiopharmaceutical production and reiterated ongoing European regulatory initiatives, in particular in view of the revision of EU`s pharmaceutical legislation. Lastly, a presentation from Maria Cojocaru-Toma at State Medical and Pharmaceutical University "Nicolae Testemitanu" updated attendees on progress in nuclear medicine and techniques using radiopharmaceutical preparations that are being implemented in the Republic of Moldova.

Unsurprisingly, regional trends in the radiopharmaceutical sciences differ quite widely around the world depending upon availability of local infrastructure and workforce, as well as variability in regulatory environment and healthcare systems between countries. Overall, however, ISTR-2023 illustrated that the field is growing more or less everywhere, from installation of new cyclotrons and scanners, to increased use of novel imaging agents and theranostics and this trend is expected to continue.

### Training and education considerations

The contributions below were either presented orally or during the poster presentations at ISTR-2023. In addition, some abstracts were submitted describing training and certification activities in Mauritius, India, and Indonesia.

Emilija Janevik-Ivanovska from Goce Delcev University presented the design, development and delivery of e-learning modules for Radiopharmacy as organized by Stip University in North Macedonia which already has over 15 years of experience with e-learning teaching. The Radiopharmacy training also includes hands-on training (to be performed in a selected host institute). The education program contains a large number of courses and is concluded by an online exam.

The EANM has a well-established educational program for radiopharmacy which is under the auspices of the Committee of Radiopharmaceutical Sciences and ESMIT. Dana Nicolae reported on the activities by EANM which include (1) symposia and CME during the EANM Annual congress, (2) Release of specific guidelines to aid in proper performance of radiopharmaceutical activities. Through ESMIT (European School of Multimodality Imaging & Therapy) postgraduate courses are organized for decades by several European centers (currently Ljubljana, Zurich and Leipzig), consisting of three theoretical blocks of two weeks each, and a practical checklist. After finishing all courses and practical activities, the EANM issues a certificate. Furthermore ESMIT organizes advanced GMP-courses which have been held 4 times so far.

A Multimodality program focusing on qualification of radiopharmacists was presented by Fabio Marques et al. of the University of Sao Paulo, Brazil. This training includes a winter school in Radiopharmacy and Chemistry (40h), a one-year on-the-job training program with extensive time in the lab (1760h), a theoretical post graduate course (90h), and a practical course (60h). The training program covers all aspects, spanning design and production of radiopharmaceuticals to translational aspects to first in man studies. The course has attracted a significant number of undergraduate and graduate students. In Brazil another training program is organized in Rio de Janeiro at the National Cancer Institute. The theoretical part comprises of 40h theory and 80h observational practice.

Mariella Teran from Universidad de la República, Uruguay, investigated virtuality as a tool to expand educational offers in radiopharmacy in South America. The study is about postgraduate training on radiopharmacy. It was concluded that virtual online tools are extremely important to facilitate access to education in radiopharmacy in South America and should be encouraged to strengthen human resources.

Two posters were submitted from Jordan dedicated to training and education. The first was from the University of Jordan dealing with the role of Pharmacists as community educators presented by Enam Khalil and colleagues. The study concluded that lack of knowledge on radiation hampered pharmacists to raise awareness on using radiopharmaceuticals and their medical applications amongst the general public. A specialized course to fill such a gap is recommended. The second contribution was presented by Derar Omari and dealt with Radiopharmacy education and regulations. Education in Jordan is organized in three sectors, academic, military and private. The academic sector comprises the Yarmouk University where radiopharmacy is included in the MSc of Pharmacy as part of a three-credit course. For the military sector, the Royal Medical Services offers a four year training and practice program, which includes preparation methods, testing, QC, and use of radiopharmaceuticals. The private sector organizes training offered by the Jordan Research and Training Reactor and created a MSc program.

Josephine Namutebi from Mbarara University of Science and Technology in Uganda submitted a poster on a survey made to assess the perception and awareness of the general public regarding the use of radiopharmaceuticals. While the population of Africa is rapidly growing, the number of nuclear medicine facilities is lagging greatly behind. For instance in Uganda there is only one nuclear medicine center. There is no training program for radiopharmacists. The survey conclusions were drawn from 80 respondents. There is an increased awareness in Uganda about the use of radiopharmaceuticals due to proper use of media platforms and promotion by youth groups.

From Thailand, a poster was presented by Putthiporn Charoenphun of Mahidol University and Nuclear Medicine Society of Thailand, on how radiopharmacy education is moving forward in this country. Thailand has 40 nuclear medicine departments and seven cyclotrons. The Nuclear Medicine Society of Thailand made an initial course based on UK, US, and EU certification systems. The course is carried by Mahidol University. Postgraduate courses are also in place. Challenges are limited career options and lack of regulations for personnel.

The take home message from the various updates provided is that, globally, training and education are urgently needed on different levels, but that initiatives are currently quite scattered. There are clear benefits to standardizing some of these efforts, and IAEA has ongoing efforts to produce training courses for its Member States in this regard.

### Women in the radiopharmaceutical sciences

Worldwide, there are a range of efforts underway to not only secure, but also diversify the radiopharmaceutical sciences workforce (Gee et al. 2020). Analia Soldati from Fundacion Instituto de Tecnologias Nucleares para la Salud gave an update specifically on women's role in radiopharmaceutical development and production in Argentina. The team explored quantitatively and qualitatively the role and situation of women in the radiopharmaceutical workforce in Argentina, using both personal interviews and literature research, and postulated the trends and necessities for equal opportunities in our field.

### Future perspectives and new initiatives

Overall the success of the ISTR shows the future of radiopharmaceutical chemistry and nuclear medicine is bright. Advances in isotope production, radiochemistry and translation of novel radiopharmaceuticals into human studies are yielding exciting results. In particular, theranostic strategies where imaging is used to direct targeted therapy and the development of new radiotherapeutics are growing areas of research that are helping the global nuclear medicine community to begin to realize precision care for personalized medicine. Additional emerging areas such as Auger emitters and the use of AI in the development of radiopharmaceuticals are on the horizon as exciting new developments. Challenges in the availability of isotopes, translation of radiopharmaceuticals into clinical trials and ultimately standard of care as well as enhancing the radiopharmaceutical development workforce remain important areas of continued focus. The ISTR is an important venue for the sharing of new techniques and enables new collaborative efforts around globe in academic, industry and federal facilities.

## Conclusions

Overall, the 3rd International Symposium on Trends in Radiopharmaceuticals (ISTR-2023) was considered a success. It was very well attended by a diverse mix of radiopharmaceutical scientists from all over the world, including many women and young investigators, and the oral and poster presentations provided a valuable update on the current state-of-the-art of the field amongst IAEA Member States. Presentations as well as networking amongst the attendees resulted in extensive knowledge transfer amongst the various stakeholders representing 88 IAEA Member States. This was considered particularly valuable for attendees from Member States where nuclear medicine and the radiopharmaceutical sciences are still relatively new. Exchanges at ISTR-2023 offered them an opportunity to learn first-hand from scientists representing commercial operations and/or research groups from around the world who have been working in the field for decades*.* Topics from the conference highlighted in this proceedings paper include: *Isotope Production and Radiochemistry, Industrial Insights, Regional Trends, Training and Education, Women In Radiopharmaceuticals, and Future Perspectives and New Initiatives.* Since the goal is for the symposium series to be held every 4 years, the next one is anticipated to take place in 2027.

## Data Availability

More details about presentations discussed in this article, as well as associated data, can be found in the Book of Abstracts available at: https://www.iaea.org/sites/default/files/23/07/cn-310_bk_of_abstracts.pdf (accessed 28th July 2023).

## Abbreviations

AI: Artificial intelligence
CNS: Central nervous system
DOE: US department of energy
EANM: European association of nuclear medicine
FDG: Fludeoxyglucose
GMP: Good manufacturing practices
HEU: Highly enriched uranium
IAEA: International atomic energy agency
ISTR: International symposium on trends in radiopharmaceuticals
LET: Linear energy transfer
LEU: Low enriched uranium
PET: Positron emission tomography
PSI: Paul Scherrer institute
PSMA: Prostate specific membrane antigen
SoS: Security of supply
SPECT: Single photon emission computed tomography
SRS: Society of Radiopharmaceutical Sciences
WHO: World health organization
